# An Intracardiac Atrioventricular Vena Caval Mass: A Case Report

**DOI:** 10.7759/cureus.45928

**Published:** 2023-09-25

**Authors:** Eric A Schafer, Marian Maldonado, Peter A Iskander, Mark M Aloysius

**Affiliations:** 1 Radiology, A.T. Still University–School of Osteopathic Medicine in Arizona, Mesa, USA; 2 Biology, Florida International University, Miami, USA; 3 Internal Medicine, The Wright Center for Graduate Medical Education, Scranton, USA

**Keywords:** cardiac sarcoma, intracaval mass, case report, cardiac tumor, intracardiac mass

## Abstract

Cardiac tumors (CTs) and intracardiac masses are rare, with an incidence of 1 per 2 million people annually. We present a case of an intracardiac mass where the patient exhibited progressive lower extremity swelling, night sweats, and diarrhea. Computed tomography of the chest with intravenous contrast revealed a sizable intracardiac mass with mixed attenuation and signs of metastatic lesions, suggesting a malignant process. This case stands out due to its uncommon presentation, considerable size, and extension from the right atrium into the right ventricle and the inferior vena cava. Although the exact etiology remains unclear because of the absence of a biopsy, it was presumed to be a type of sarcoma. Owing to significant cardiac obstruction, the patient's condition worsened rapidly, culminating in a fatal outcome mere days after the initial presentation. While there are multiple approaches to identify and treat CTs, their propensity to grow quietly until they reach a size large enough to cause fatal symptoms restricts opportunities for early detection and treatment.

## Introduction

Intracardiac masses are most commonly caused by thrombi, hamartomas, structural abnormalities, and infectious vegetations. Neoplastic causes are rare and can arise from metastatic disease or a primary etiology. The most common primary neoplastic masses are myxomas/papillary fibroelastomas (PFEs) in adults and rhabdomyomas and fibromas in the pediatric population [1]. While most cardiac masses are found incidentally and without symptoms, some may produce enough mass effect to cause structural issues. Evaluating an intracardiac mass involves many factors including age, clinical picture, localization, and imaging. Characterization of masses includes transesophageal echocardiography (TEE), magnetic resonance imaging (MRI), and computed tomography (CT). Novel techniques in detection include three-dimensional echocardiography, which can achieve better visualization of vascularity, homogeneity, point of attachment, and calcification [2]. The distinctiveness of this case arises from the characteristics of the mass, including its substantial dimensions, its projection from the right atrium into the right ventricle, and its contiguous connection with the inferior vena cava.

## Case presentation

A 69-year-old female with a 40-pack-year smoking history and asthma presented to the emergency room with complaints of progressive bilateral lower extremity edema, diarrhea for three weeks, and night sweats for a month. She denied any fever, chills, chest pain, shortness of breath, nausea, or dysuria. She had a chronic cough that was unchanged from baseline, which was occasionally productive without hemoptysis. She denied any known history of cardiac or gastrointestinal disease. She had not been receiving any routine medical care for the past 10 years due to being uninsured. She was not on any medications at the time. On presentation, she was ill-appearing and had 4+ bilateral pitting edema. Her vitals were tachycardic to 109 beats per minute but otherwise stable. Initial lab work showed leukocytosis of 17,900/mm^3^, hemoglobin 10.2 g/dL, hematocrit 32%, sodium 130 mEq/L, lactic acid 2.3 mg/dL, and a brain natriuretic peptide 1120 pg/mL. Chest X-ray showed multiple bilateral pulmonary nodules and small bilateral pleural effusions with atelectasis. Chest CT with intravenous contrast revealed a large mass extending from the inferior vena cava (IVC) into the right atrium and right ventricle. It measured up to 4 cm in the right atrium and 7 cm in the right ventricle and is of mixed attenuation. There were also numerous pulmonary and hepatic parenchymal nodules up to 1 cm in size suspicious for metastatic disease. The patient was admitted to the medical intensive care unit on empiric intravenous antibiotics, pressors, and diuretics. Over the next three days, she deteriorated with presumed obstructive shock and multiorgan failure. Given the size of the tumor, extension, and metastasis, the tumor was deemed inoperable. The patient's family subsequently withdrew life-sustaining support. She passed away four days later. An autopsy was not performed.

## Discussion

Certain radiological features of intracardiac masses can help distinguish their etiology. This includes size, location (left vs right), morphology, attachment, contrast enhancement, margins, invasion, metastasis, history of pericardial effusion, and calcification. The details of these features are featured in Table 1 [3]. 

**Table 1:** Cardiac CT features of benign and malignant tumors

Feature	Benign	Malignant
Size/number	Small (<5 cm), single lesion	Large (>5 cm), multiple lesions
Location	Left >> Right	Right >> Left
Morphology	Intracameral	Intramural
Attachment	Narrow stalk, pedunculated	Broad base
Enhancement	Absent to minimal	Modest to intense
Margin	Smooth, well-defined	Irregular, ill-defined
Invasion	None	Intra-/extracardiac infiltration
Metastasis	None	May be present
Pericardial effusion	None	May be present
Calcification	Rare (except for small foci in fibroma, myxoma, or teratoma)	Large foci in osteosarcoma

**Figure 1:**
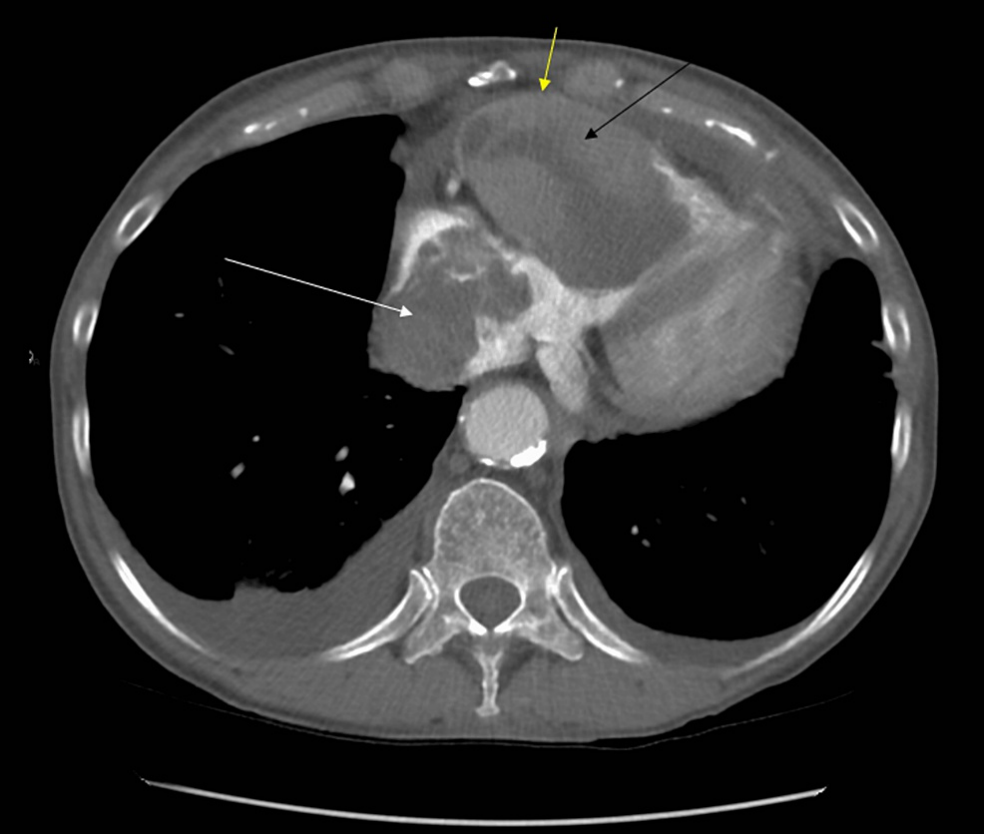
CT chest with contrast in the transverse plane. There is a large mass of mixed attenuation within the right ventricle (black arrow). There is an extension of the mass into the right atrium that also contains a mixed attenuation with likely thrombus formation (white arrow). There is no contrast separating the ventricular mass from the wall, likely indicating invasion or attachment to the ventricular wall (yellow arrow).

**Figure 2:**
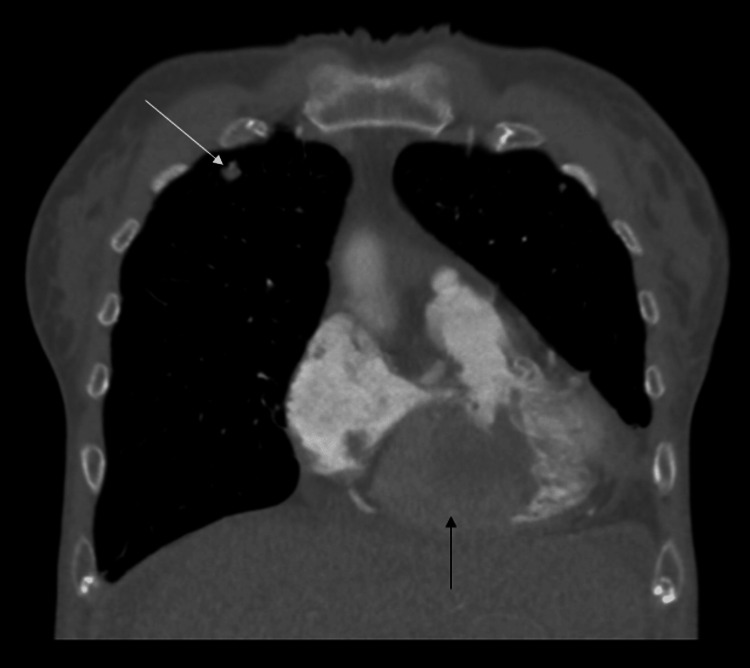
CT chest with contrast in the coronal plane displaying the right ventricular mass (black arrow) and right upper lobe nodule (white arrow).

**Figure 3:**
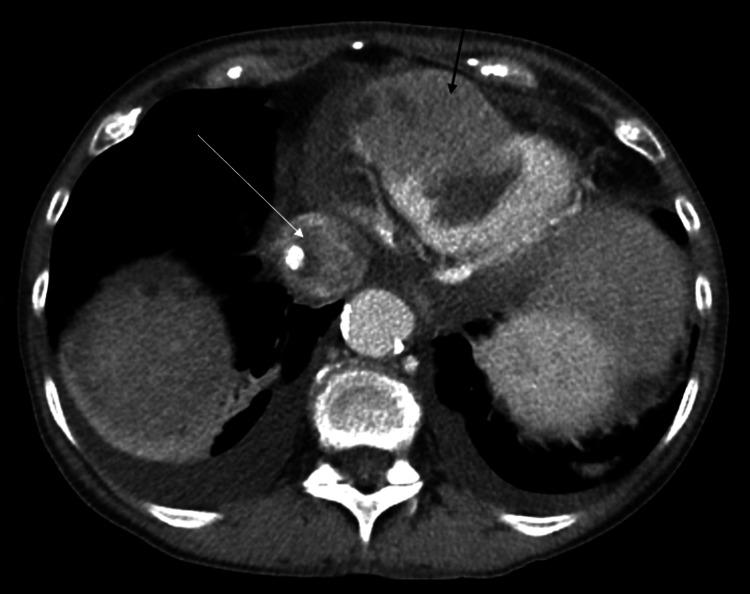
CT Abdomen with contrast in the transverse plane showing the cardiac mass with mixed attenuation in the right ventricle (black arrow) as well as within the inferior vena cava (white arrow).

**Figure 4:**
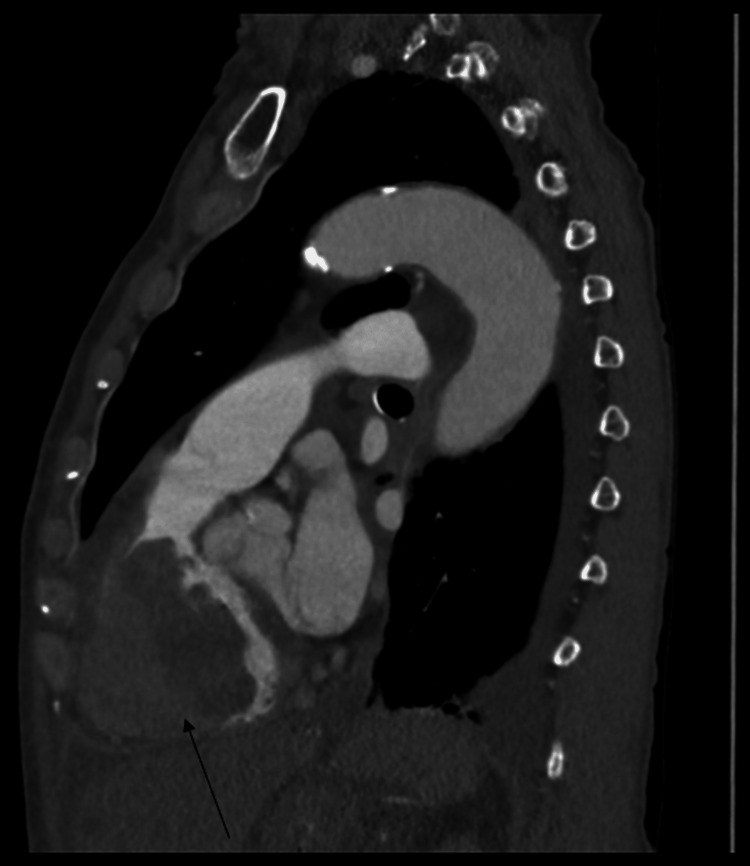
CT chest with contrast in the sagittal plane showing a large intracardiac mass within the right ventricle (black arrow).

**Figure 5:**
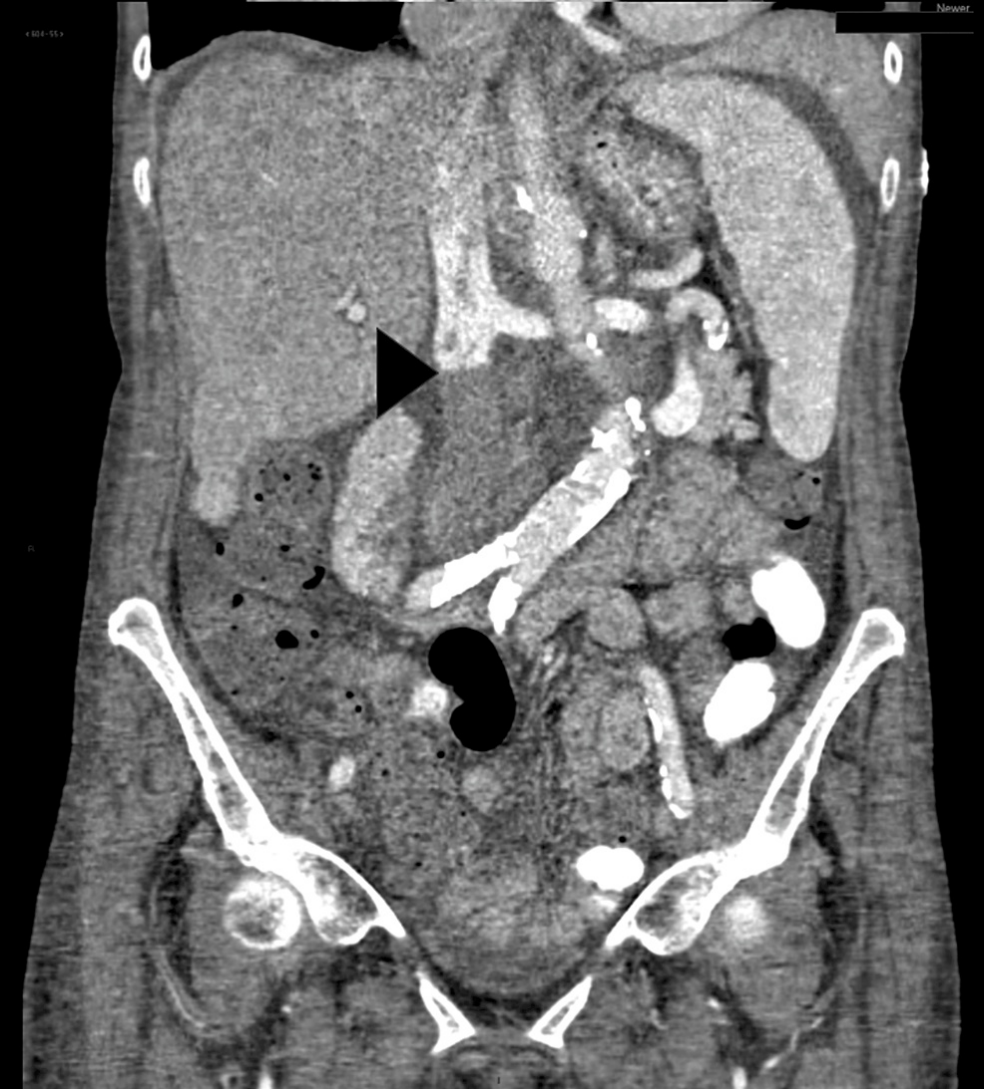
CT abdomen with contrast in the coronal plane showing a large thrombus completely occluding the inferior vena cava (black arrowhead) as well as mass/thrombus extension proximally.

In this particular case, the mass is large (7 x 3.5 cm at its largest within the right ventricle, Figure 1), and it is present within the right atrium and ventricle and extends into the IVC (Figures 3, 4, 5). It is intramural and without evidence of a stalk. There is evidence of contrast enhancement with central areas of hypoenhancement. There are poorly defined margins without clear evidence of invasion. There are multiple metastatic nodules up to 1 cm in size in the lungs (Figure 2) as well as a hepatic nodule (not pictured). There is no large pericardial effusion or calcifications. These findings are suggestive of a malignant etiology.

General principles guiding the decision for surgical excision of CTs revolve around several factors, including the potential for malignancy, clinical manifestations (e.g., obstructive shock), and overall ability to tolerate surgery. The *Journal of the American College of Cardiology *(JACC) CardioOncology state-of-the-art review classifies the role of surgery between different types of CTs. For primary benign CTs, such as lipomas and rhabdomyomas, surgical intervention is only recommended in severely symptomatic cases. Certain primary benign CTs are prone to other complications, including embolic events in myxomas and papillary fibroelastomas, sudden cardiac death in infants with fibromas, and catecholamine surges in paragangliomas. These complications warrant surgical resection where possible. Primary malignant CTs, such as leiomyosarcomas, rhabdomyosarcomas, angiosarcomas, osteosarcomas, undifferentiated sarcomas, and mesotheliomas, are prone to invasion metastasis and paraneoplastic complications; thus, they warrant surgical resection when possible. One notable exception is primary cardiac lymphoma, which is treated with anthracycline chemotherapy and anti-CD20 monoclonal antibody treatment rituximab. Metastatic tumors within the cardiac chambers are typically indicated for surgical removal due to their potential for local invasion and obstructive tendencies [4]. 

Due to their rarity in incidence, large-scale trials have not been performed on many of the primary cardiac malignant tumors. Systemic therapy used in a previous study involving primary cardiac lymphoma includes the use of Anti-CD-20 drugs (e.g., rituximab) and anthracycline therapy (e.g., doxorubicin) with relatively high response rates (62% complete response and 23% partial responses) but with high recurrence (55%) [5]. Response rates were measured using the Cheson revised criteria, which define complete response as the disappearance of all evidence of disease including clinical evidence and disease-related symptoms present before therapy. Partial response is defined as at least a 50% decrease in the sum of product diameters of up to six of the largest masses or nodes [6]. 

Besides primary cardiac lymphoma, a glaring issue in many of the studies involving CT response to chemotherapy is the lack of adequate sample size and power. Pediatric rhabdomyosarcoma, which benefits from a combined regimen of surgery, systemic chemotherapy (vincristine, dactinomycin, and cyclophosphamide), and radiotherapy, accounts for 4%-7% of cardiac sarcomas, but are also highly invasive and often fatal within a year [4]. 

Survival rates of CTs are generally poor, regardless of origin. A retrospective study on the incidence and survival of primary CTs found a five-year survival rate of 14% [4]. A large multi-institute retrospective study utilizing the national cancer database observed the outcomes of 747 cases of primary CTs and found a five-year survival rate of 11.5% [7]. 

## Conclusions

CTs are rare diagnoses with poor prognosis secondary to obstructive shock and fatal arrhythmias. The use of multiple imaging modalities such as TEE, MRI, CT, and 3D echocardiography has played a large role in identifying tumors and guiding the approach to therapy. Treatment options include surgical resection, systemic chemotherapy, and radiation. While small CTs may be clinically benign, larger tumors can be fatal, without much improvement in survival after multifactorial treatment. With this case, we hope to add to the existing literature on these rare tumors, and further longitudinal studies are needed to study and describe their behaviors. 
